# Amyloid structure exhibits polymorphism on multiple length scales in human brain tissue

**DOI:** 10.1038/srep33079

**Published:** 2016-09-15

**Authors:** Jiliang Liu, Isabel Costantino, Nagarajan Venugopalan, Robert F. Fischetti, Bradley T. Hyman, Matthew P. Frosch, Teresa Gomez-Isla, Lee Makowski

**Affiliations:** 1 Department of Bioengineering, Northeastern University, Boston, MA, USA; 2 Massachusetts Alzheimer’s Disease Research Center, Massachusetts General Hospital, Boston, MA, USA; 3 GM/CA@APS, Argonne National Laboratory, Illnois 60439, USA.

## Abstract

Aggregation of Aβ amyloid fibrils into plaques in the brain is a universal hallmark of Alzheimer’s Disease (AD), but whether plaques in different individuals are equivalent is unknown. One possibility is that amyloid fibrils exhibit different structures and different structures may contribute differentially to disease, either within an individual brain or between individuals. However, the occurrence and distribution of structural polymorphisms of amyloid in human brain is poorly documented. Here we use X-ray microdiffraction of histological sections of human tissue to map the abundance, orientation and structural heterogeneities of amyloid. Our observations indicate that (i) tissue derived from subjects with different clinical histories may contain different ensembles of fibrillar structures; (ii) plaques harboring distinct amyloid structures can coexist within a single tissue section and (iii) within individual plaques there is a gradient of fibrillar structure from core to margins. These observations have immediate implications for existing theories on the inception and progression of AD.

Aggregates of Aβ peptides in the brain are key pathological features of Alzheimer’s Disease (AD)^1^, but their role in disease remains under debate^2^,^3^ in part because the level of Aβ plaques does not correlate well with severity of cognitive decline^4^ and build-up of amyloid plaques may precede symptoms by years^5^,^6^. This lack of correlation might be explained if soluble oligomers of Aβ, rather than fibrils are responsible for synaptic and neuronal damage in AD^7^,^8^,^9^. Furthermore, it may be that different structural forms of fibrils are associated with different disease states^10^,^11^.This hypothesis is supported by observations that amyloid generated *in vitro* by assembly from two patients with distinct clinical histories exhibited different structures as determined by ssNMR^12^. Individuals asymptomatic for dementia despite substantial AD pathology (mismatch cases) may also harbor atypical amyloid as suggested by relatively low levels of plaques that react with fibrillar thioflavin-S or with a conformer-specific antibody as compared to demented AD subjects^13^.

The suggestion that different amyloid structures may contribute differentially to disease seems to imply the existence of a single predominant polymorph within a brain^12^, at odds with the widespread observation of structural polymorphism in fibril structure that can be seen within a single preparation^14^,^15^,^16^,^17^. Fibrils formed from synthetic Aβ(1–40) display a baffling range of morphologies^18^,^19^,^20^. The predominant structure can be influenced by a wide range of modulating factors^21^ and controlled by subtle variations in fibril growth conditions^22^,^23^. Fibrils have been observed to undergo conformational rearrangements during long term storage^24^ and during aging in animal models^25^. Although self-propagation of amyloid fibrils appears to preserve distinctions among structure types^12^,^22^ the possible impact of conformational selection during assembly has not been determined^26^. The ensemble of fibril structures in a sample almost certainly depends on both environment and history. But the extent to which a single fibril structure predominates in a single brain *in vivo* is unclear and the impact that different predominant structures may have on eventual clinical presentation remains unknown.

Detailed study of synthetic Aβ(1–40) fibrils *in vitro* has the potential to provide a basis for understanding the behavior of amyloid *in vivo*, but the relationship between *in vitro* assembled fibrils and fibrils active in disease is uncertain^17^. Characterization of amyloid fibrils formed *in vitro* can take advantage of a wide range of biophysical techniques and has resulted in the creation of dozens of structures and structural models. But only a limited number of methods can carry out analyses of fibrils *in situ*. As we will report here, X-ray microdiffraction is amenable to *in situ* studies because it utilizes a micron-sized x-ray beam to collect data from very small scattering volumes making possible the targeting of regions with high amyloid concentration and low abundances of other constituents. Here, we use it to generate detailed structural information about amyloid fibrils *in situ*, map the spatial distribution of structural heterogeneities and identify differences in amyloid structure from subjects with different clinical histories.

X-ray scattering from amyloid fibrils is largely confined to equatorial scattering in the small angle and ~10 Å regimes; and meridional scattering in the wide angle regime - in particular the strong ~4.7 Å layer line which, because of poor orientation is often reported as a single reflection. The combination of equatorial 10 Å peak and meridional 4.7 Å peak was used to make the original assignment of a cross-β structure to amyloid^27^,^28^. This general pattern of scattering has been observed in a wide variety of amyloid preparations both natural and synthetic^29^, including Aβ fibrils formed *in vitro*^30^; scrapie protein rods^31^; natural and synthetic prion structures^32^; and many others^33^ and is considered a diagnostic for the presence of amyloid. Variation in the observed intensities provides information about differences among amyloid preparations. For instance, the Iowa mutation results in fibrils that exhibit a shift in the position of the ~10 Å peak compared to WT Aβ(1-40) fibrils^34^, indicating the average spacing between layers of β-sheets is smaller in D23N-Aβ40 than in WT-Aβ40. Small-angle equatorial data contains information about the cross-sectional size and shape of the fibrils and was used to evaluate and refine molecular models of fibril structure originally constructed on the basis of ssNMR data^35^.

The shape and breadth of the 4.7 Å peak is highly variable^36^. It provides information about the arrangement of Aβ peptides within the fibrils. For instance, in fibrils formed from Aβ(1–40) the breadth of the peak varies dramatically between fibrils formed at pH 2 and at pH 7.4 (Fig. 1 in Macdonald *et al*.^35^). Although amyloid structure has been reported to be unchanged by dehydration^37^, data reported in that paper show, on dehydration, a slight shift of the 4.7 Å peak to higher scattering angle - indicating a small decrease in the average inter-strand spacing. In a study of six different *ex vivo* and two synthetic amyloid fibril preparations Sunde *et al*.^33^ observed that the ~4.7 Å peak always appears as a doublet, with peaks at roughly ~4.82 Å and ~4.63 Å, although in some cases the 4.63 Å peak was sufficiently weak to be observed only as a shoulder on the stronger 4.82 Å peak. Blake and Serpell^38^ observed the ~4.7 Å peak in transthyretin amyloid to be a doublet with peaks at 4.83 Å and 4.64 Å. They interpreted the doublet as the 24th and 25th orders of an ~115Å axial repeat^33^,^38^ which, although consistent with the expected properties of β-sheets^39^,^40^, is inconsistent with cross-over separations often observed by EM^14^.

A more likely interpretation of the ‘doublet’ nature of the 4.7 Å peak is that it represents a visualization of a pair of reflections on the 4.7 Å layer line. Because of poor orientation of fibrils, the intensity along the layer line is arced about the center of the pattern transforming distances along a layer line into apparent radial separations. The lower angle peak (~4.75 Å) corresponds to a meridional or near-meridional reflection; the wider angle peak (~4.65 Å) to an off-meridional reflection^41^. Jahn *et al*.^36^ explored how observed diffraction patterns can differ from those calculated from ‘idealized’ amyloid models and reported differences in the ratio of 4.75/4.65 intensity ratios. The near universal observation of these two peaks in scattering from a dramatic variety of amyloids may imply a limit to the number of possible quaternary structures consistent with structural stability of the fibrils. Small differences in twist or tilt of the peptide subunits will give rise to observable changes in the relative intensities of these peaks. These differences may be common because small adjustments of tilt or twist are unlikely to be resisted by high energy barriers (e.g. Fig. 2 in Fandrich *et al*.^14^). Larger changes in the relative intensities may not be explained by subtle changes in tilt or twist and may be associated with quaternary re-arrangements of subunit peptides.

In order to assess the degree of polymorphism present in amyloid fibrils *in situ*, we used X-ray microdiffraction of histological sections of human tissue to map the abundance, orientation and structural heterogeneities of amyloid within individual plaques; among proximal plaques and in subjects with distinct clinical histories.

## Results

### *In situ* Microdiffraction of Amyloid in Human Brain Tissue

Brain tissue from the amygdala of three AD subjects; one mismatch case, two age-matched controls and a subject diagnosed as having Pick’s disease were processed by standard histological methods (see Methods section) and spread onto 12 μ thick mica films to act as substrates to hold the samples in place for x-ray scattering. Serial sections, immediately adjoining the x-ray samples were captured on glass microscope slides and immunostained for presence of Aβ peptides. In all cases care was taken to collect x-ray data from regions expected to have highest plaque burden on the basis of microscopic examination of the immunostained sections since scanning microdiffraction cannot be readily used to survey large regions of tissue. For the age-matched controls and Pick’s subject no evidence of Aβ peptides was observed with immunostaining.

Presence of amyloid was judged on the basis of the observation of a sharp 4.7 Å reflection known to be characteristic of cross-β structure. The proportion of diffraction patterns exhibiting this signature reflection was highly variable among fields of view even in the same subject. The number of diffraction patterns exhibiting this reflection is reported for each subject in Table 1. In one field of view, over 350 of 2500 diffraction patterns exhibited this reflection. In contrast, in one age-matched control no 4.7 Å reflection was detected in any of the 2500 diffraction patterns collected. In the other control, 2 of 2500 diffraction patterns had weak scattering at 4.7 Å spacing with characteristics indicating the presence of amyloid. A characteristic of Pick’s disease is the presence of neurofibrillary tangles (NFTs) - deposits of tau protein in the absence of amyloid. NFTs might be expected to give rise to sharp reflections in the 4.7 Å region if they have a cross-β structure. Interestingly, only four patterns with evidence of weak 4.7 Å reflections were observed in scattering from the Pick’s disease subject, leaving open the issue of identifying NFTs by microdiffraction. A summary of all fields of view collected is included in Table 1. A total of 772 diffraction patterns that contained a sharp 4.7 Å reflection were recorded out of a total of 22,800 patterns collected.

**Figure 1:**
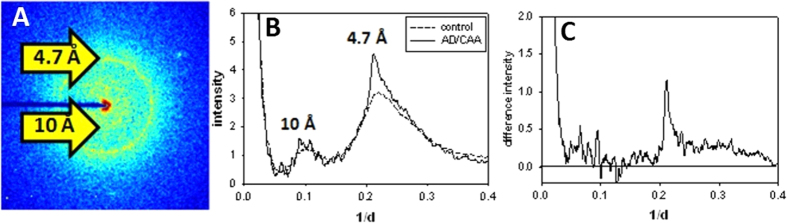
X-ray microdiffraction from thin sections of human brain tissue: (**A**) X-ray scattering pattern from a thin section of human AD brain tissue after subtraction of background scattering from mica substrate and air. A sharp reflection from amyloid at ~4.7 Å spacing can be seen as a yellow ring. Diffuse scattering in the 10 Å region can be seen as a yellow halo closer to the center. The dark blue bar to the left is a shadow of the beam stop holder. (**B**) Circularly averaged intensity of a scattering pattern from tissue containing an amyloid plaque (solid line) and a control region without amyloid (dashed line). (**C**) Scattering from plaque minus control region.

Figure 1 is representative microdiffraction scattering data from an unstained 18 μ thick section of human brain tissue taken from an AD subject with typical Aβ distribution and progression of cognitive decline. Figure 1A is a detector image (after subtraction of background due to scattering from the mica substrate and air) from a region identified as harboring an amyloid plaque based on the presence of a strong, sharp 4.7 Å scattering peak. The sharp 4.7 Å peak is clearly visible along with diffuse scatter at a spacing of ~10 Å and strong small angle scattering. Figure 1B is a plot of the circularly averaged intensity from a region containing amyloid and exhibiting a 4.7 Å peak (solid curve) and from a proximal (control) region devoid of amyloid and exhibiting only diffuse scatter at 4.7 Å spacing (broken curve). Figure 1C is a trace of the difference between scattering from a region containing amyloid and a proximal control region. This difference intensity appears typical of regions we identify as having high amyloid content and includes strong small angle (SAXS) scattering, high noise levels in the ~10 Å region and a sharp reflection centered at roughly 4.7 Å spacing. The broad peak centered at roughly 4.7 Å appears typical of microdiffraction from a broad range of tissue devoid of amyloid and processed by standard histological methods. For instance, the diffuse scatter is remarkably similar to that reported for kidney tissue prepared by similar histological methods^42^ and presumably reflects average properties of tissue that has undergone this treatment. The diffuse scatter does not exhibit orientation and does not vary markedly in shape from place to place in a single section. It does, however, vary in intensity and this variation provides some measure of the mass of amorphous tissue remaining in the section after histological processing, making possible the identification of landmarks such as small blood vessels. These features aid the alignment of x-ray scans to optical images collected from serial sections.

**Figure 2:**
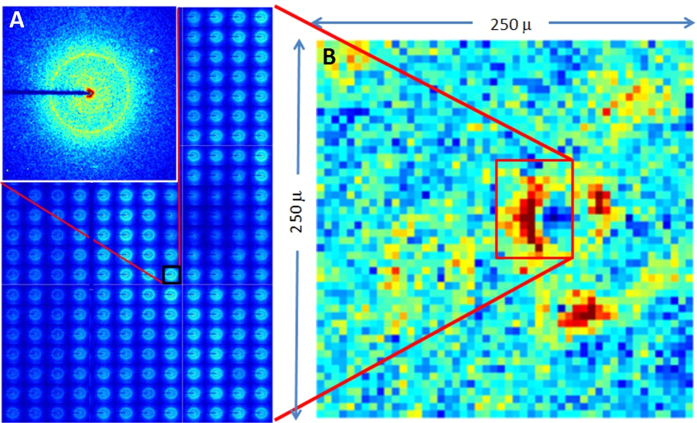
Scanning microdiffraction data set. (**A**) A single diffraction pattern from an amyloid containing region superposed on a montage of 180 diffraction patterns, part of 2500 diffraction patterns collected from a section of AD brain tissue. (**B**) Mapping of the sharp ~4.7 Å scattering intensity in 2500 patterns collected on a 5 μ grid. Dark blue corresponds to weak scattering; dark red, the strongest 4.7 Å intensity. Field of view includes two amyloid plaques in the margin of a blood vessel, a third plaque 40–50 μ below it and two regions of diffuse amyloid in the upper corners of the field of view.

The distribution of amyloid across a 250 × 250 μ field of view was mapped by collecting 2500 diffraction patterns using a 5 μ beam scanned over a 5 μ grid and automatically capturing the intensity of the sharp 4.7 Å reflection in each pattern. Figure 2A displays a single diffraction pattern from a scan of a typical AD subject and a montage containing 180 of the 2500 diffraction patterns collected in the scan. The distribution of amyloid in this field of view was mapped by integrating the intensity of the sharp 4.7 Å peak and displaying it coded by color in Fig. 2B. Intensity estimates for the 4.7 Å peak were made by fitting a polynomial to the diffuse background and integrating the intensity above that background in the vicinity of the 4.7 Å peak. Color coding in Fig. 2B was set so that the lightest orange corresponds to the level of noise judged to be the threshold above which the presence of a sharp 4.7 Å reflection was likely indicative of amyloid. In this field of view, amyloid (bright orange/red) appears to be concentrated in two regions proximal to a small blood vessel and a third region about 50 μ from the margin of the blood vessel. A region in the extreme upper left and another near the upper right corner of this field of view may correspond to small, perhaps relatively diffuse deposits of amyloid. Scattering from any additional amyloid in this field of view is at or below the threshold necessary for confident identification.

**Figure 3:**
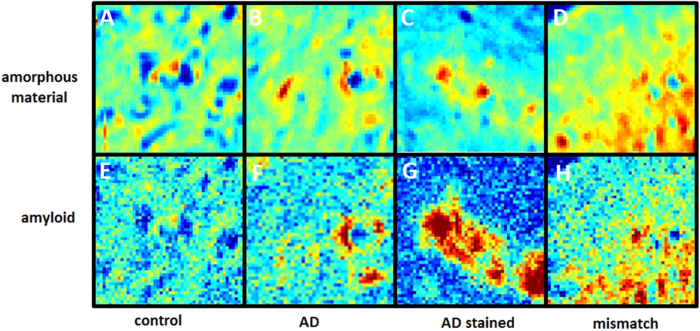
Distribution of intensity in 10,000 diffraction patterns collected from 250 × 250 μ fields of view in thin sections of human brain tissue. Each pixel in these images represents the average intensity from one diffraction pattern as calculated from the (top) amorphous scattering in the 4.7 Å region and (bottom) the sharp 4.7 Å reflection from amyloid. Data from samples that include (**A,E**) an age-matched control; (**B,F**) typical AD case; (**C,G**) typical AD case stained with Congo Red; and (**D,H**) mismatch case. Diffuse scattering intensity (**A–D**) provides an overall visualization of the distribution of cellular material in each thin section. Dark blue corresponds to regions of very low density including small blood vessels; red corresponds to relatively high density regions. Location of amyloid deposits are indicated by dark red in the lower panels.

Figure 3 shows the distribution of amorphous material (A–D) as determined by the intensity of diffuse, wide-angle scattering; and distribution of amyloid (E–H) as estimated from the intensity of the sharp 4.7 Å reflection (after subtraction of the diffuse scatter - see Fig. 1). Data are shown for sections from (A,E) an age-matched control subject that had no signs of dementia at time of death; (B,F) a typical AD subject without and (C,G) with Congo Red (CR) dye staining; and (D,H) a mismatch case. In the control, amorphous tissue density appears greatest between two small blood vessels near the center of the field of view. In that region the automatic processing of data detected the presence of a weak, sharp 4.7 Å peak that is at or slightly above the threshold level of detection, suggesting the possibility of a small amyloid deposit. The unstained AD sample exhibits at least three well-formed plaques (two proximal to a small blood vessel) and two additional regions that harbor diffuse amyloid deposits. In the stained tissue sample, several large plaques are observed. The binding of CR dye appears to enhance scattering intensity in the 4.7 Å peak. In the mismatch case at least four plaques can be observed in the lower right quadrant of the scan.

Scanning microdiffraction was carried out on Congo Red stained tissue from an AD subject. Congo red dye binds specifically to amyloid and has been used for years as a stain for locating regions with high amyloid concentration. Supplementary Material contains an optical micrograph of a Congo Red stained 18 μ thick section and mapping of the distribution of intensity in the sharp 4.7 Å reflection across the same 250 × 250 μ field of view. The observation of a sharp 4.7 Å peak is largely confined to the stained region, supporting the identification of the sharp 4.7 Å peak as being due to amyloid.

Scans of tissue from two additional AD cases are contained in the Supplementary Material. In the first, a single plaque was observed along with several positions that may contain sporadic amyloid. In the second, a blood vessel dominated the field of view and the only diffraction patterns exhibiting sharp 4.7 Å reflections were isolated and showed no indication of organization into plaques. A second scan of the mismatch case (see supplementary material) appears to include a pair of diffuse plaques that may indicate the presence of a relatively large, low density aggregation of amyloid in the lower left quadrant. A second scan of an age-matched control (Supplementary Material) is essentially devoid of any evidence of amyloid.

A scan of tissue from a Pick’s disease subject that exhibited no sign of Aβ peptides but abundant presence of Tau by immunohistology was carried out in an attempt to identify the diffraction signature of neurofibrillary tangles. However, the scan showed scant evidence of a sharp 4.7 Å peak in only 4 of 2500 diffraction patterns, providing no substantive information about the nature of scattering from tangles in these samples.

### Fibril Orientation

**Figure 4:**
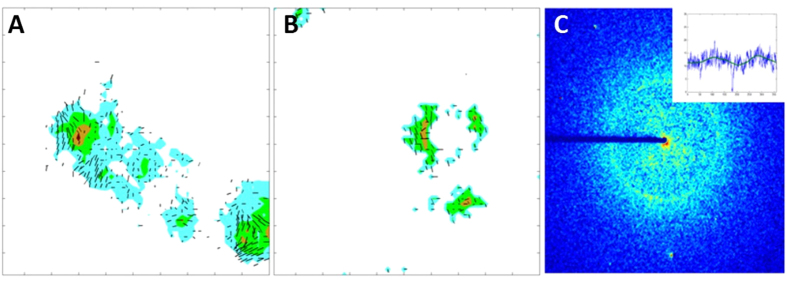
Mapping the orientation of fibrils in plaques. The average direction of orientation of fibrils (as indicated by dark lines - longer lines indicate better orientation) is overlaid on a mapping of the intensity of the 4.7 Å peak (indicated by colors with blue, green, brown and red progressively indicative of stronger scattering). (**A**) CR stained tissue from an AD subject. (**B**) Unstained tissue from an AD subject. (**C**) X-ray pattern from region exhibiting modest orientation – inset is intensity of 4.7 Å scattering as a function of angle about the center of the pattern.

In many of the diffraction patterns harboring a sharp 4.7 Å peak, there is variation in the 4.7 Å peak intensity as a function of angle about the center of the diffraction pattern. In these cases, the angle at which the highest intensity of the peak occurs indicates the most common orientation of fibrils in the scattering volume. In Fig. 4 the 4.7 Å intensity in two of the fields of view shown in Fig. 3 are re-mapped as colored contours indicating the intensity of the scattering. Superimposed on this mapping is a set of dark lines indicating the direction and degree of orientation of amyloid within the plaques. Longer lines indicate more pronounced orientation. Orientation of fibrils is correlated over distances of 20–30 μ or more and, at the margins of the plaques, may be either radial or tangential showing more diversity of organization than suggested by optical methods^43^. Lack of orientation at the center of the plaque may reflect disorganization, may be due to superposition of distinct oriented layers at different levels within the section, or may reflect orientation of the fibrils in the direction parallel to the x-ray beam (the direction perpendicular to the tissue section) which is not observable. The observation of orientation is limited by signal to noise ratio in the data. The inset (upper right corner) indicates the azimuthal distribution of intensity, with intensity in the direction of orientation only about 20% greater than in the orthogonal direction. Because of the stronger intensity of scatter in the CR-labeled samples, the apparent orientation may appear overestimated compared to the unlabeled sample. Signal-to-noise ratio in the mismatch case was inadequate to unambiguous map orientation except at sporadic positions across the fields of view. The combination of disoriented core and oriented periphery seen in this Figure is reminiscent of the organization of fibrils in plaques as deduced by optical methods^43^,^44^.

### The 4.7 Å doublet

**Figure 5:**
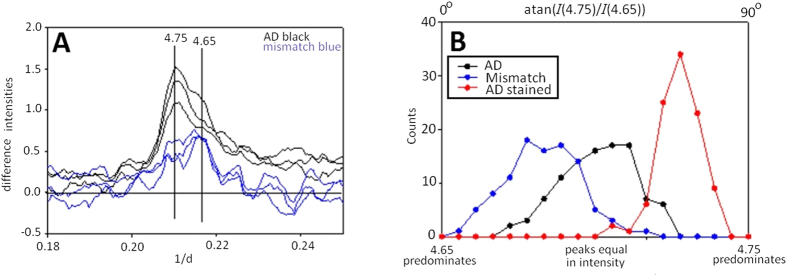
Shape of the 4.7 Å reflection from amyloid plaques in a typical AD subject and in a mismatch case. (**A**) The 4.7 Å peak appears as a doublet and the ratio of intensity at ~4.75 Å and 4.65 Å is variable, even within a single amyloid plaque. The variation in ratio is evident in the differences among the three patterns shown from a typical AD subject (black). One pattern has a significantly higher 4.65 Å shoulder than the other two. In scattering from amyloid in the mismatch case (blue), the ratio of intensities is reversed, indicating a different structural organization. (**B**) The ensemble of structures in the mismatch case is significantly shifted in histograms of the ratio of intensities [plotted as a function of atan(*I*(4.75 Å)/*I*(4.65 Å)) - angle whose tangent is the ratio of the intensities of the 4.75 Å and 4.65 Å peaks] for the strongest 4.7 Å peaks in the AD case (black), mismatch (blue) and compared to the Congo Red stained AD case (red). Staining with Congo Red results in a significant strengthening of the 4.75 Å peak relative to the 4.65 Å shoulder.

**Figure 6:**
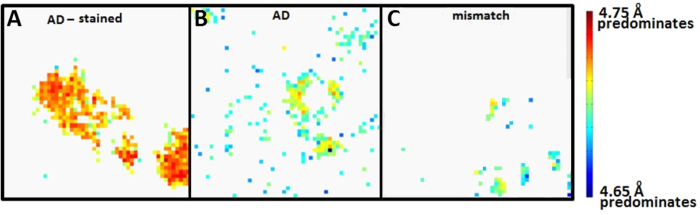
Mapping of ratio of intensity in the 4.75 Å and 4.65 Å peaks in (**A**) a typical AD subject - section stained with Congo Red; (**B**) a typical AD subject with a small blood vessel and no staining; and (**C**) a mismatch case. Color is coded as atan(*I*(4.75 Å)/*I*(4.65 Å)) [angle whose tangent is the ratio of the intensities of the 4.75  Å and 4.65 Å peaks] with red corresponding to 4.75 Å dominating; blue to 4.65 Å dominating and green to equal intensities. 250 × 250 μ fields of view. Regions without color (gray background) are regions with no detectable 4.7  Å peak (intensity of Gaussian peak less than 3 σ- see Methods).

The 4.7 Å peak usually appears as a doublet as seen in Fig. 5A. In scattering from the plaques in the AD subject, the intensity of the smaller angle peak (4.75 Å) is usually greater than that of the wider angle (4.65 Å) peak, which often is observed as a shoulder of the more intense peak. [Although the positions of the two sub-peaks vary somewhat from pattern to pattern, we will refer to their positions as 4.75 Å and 4.65 Å for simplicity]. The ratio of the intensities of these two peaks was estimated by fitting the intensity in the 4.7 Å peak to a pair of Gaussians. In most diffraction studies of amyloid where the doublet has been observed, the 4.75 Å peak is of greater intensity than the 4.65 A peak^36^. In scattering from tissue of the mismatch case, the ratio of intensities in the 4.75 and 4.65 Å sub-peaks is often inverted compared to typical AD. This can be seen in the blue traces in Fig. 5A and in the histograms in Fig. 5B. Staining with Congo Red greatly enhances the intensity of the 4.75 Å peak relative to the 4.65 Å peak. This can be seen in the histograms in Fig. 5B which document the distribution of the ratio for three samples. The histograms document a significant variation in the ratio within a single sample. The spatial distribution of regions with different ratios, as plotted in Fig. 6 indicates that this variation is not random, but varies systematically with position. This figure emphasizes both the variation among samples and within samples. The highest ratio (indicated as red in Fig. 6) being seen only in the Congo Red-stained sample and the lowest ratio (shown as blue) being largely confined to the mismatch case. The AD case exhibits an interesting variation in ratio: Amyloid with the greatest 4.75/4.65 ratio is found near the cores of plaques - both perivascular and otherwise - while amyloid with lower ratio appears more common at the margins of plaques or in isolated positions where estimation of the ratio was sporadically possible away from the more organized plaque regions. The mismatch case appears to contain plaques with the inverted ratio but also contains plaques with ratios approaching that observed for typical AD cases. This can be seen in the scan in Fig. 6C where the cores of two small plaques have a ratio similar to that typical of the AD field of view in Fig. 6B, whereas two others have an inverted ratio.

**Figure 7:**
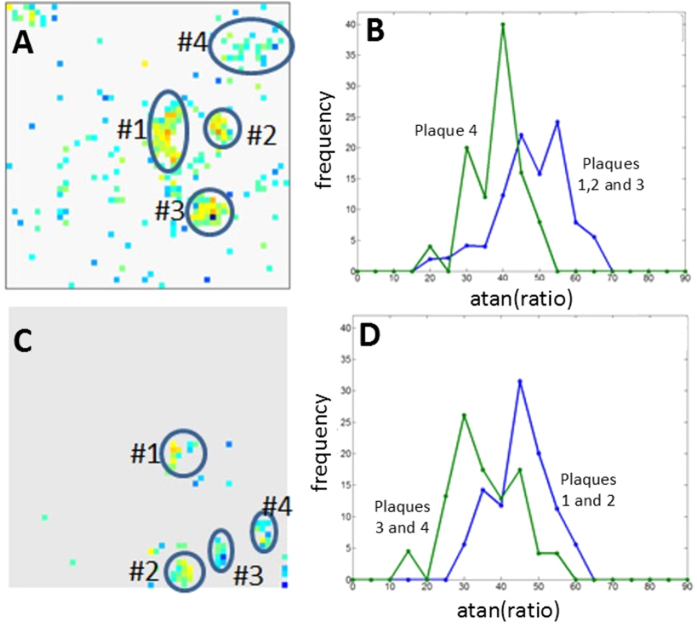
Mapping of ratio of intensity in the 4.75 Å and 4.65 Å peaks in (**A**) a typical AD subject with a small blood vessel and (**C**) a mismatch case. (**B**) contains the average of histograms of the three densest plaques from the AD field of view in (**A**), compared to the histogram of the diffuse amyloid region (labeled #4). Although there is substantial overlap of the histograms, the 4.75 Å sub-peak dominates in the majority of the dense plaques (*I*(4.75 Å)/*I*(4.65 Å) > 1.); the 4.65 Å sub-peak dominates in the diffuse amyloid (*I*(4.75 Å)/*I* (4.65 Å)) < 1. (**D**) contains a histogram of the two diffuse plaques (#3 and #4) in the mismatch case compared to that of the denser plaques observed in this field of view (#1 and #2).

In both AD and mismatch cases, there are variations in fibril structure between plaques in the same tissue section. Figure 7A,B, from a typical AD case, compares the structure of the 4.7 Å peak in two classes of plaques which exhibit a significant difference in their histograms. The regions with the smallest ratio of 4.75 to 4.65 Å sub peaks are found in the diffuse amyloid at the margins or external to the plaques. The plaque with the smallest 4.75/4.65 ratio appears more diffuse than the others, reflecting that trend. Figure 7C,D compares two classes of plaques in the mismatch case which appear distinct from one another but also constitute an ensemble of structures with peak shapes shifted relative to that observed in tissue from the typical AD subjects.

## Discussion

Here we have presented evidence of the existence of structural polymorphisms in amyloid fibrils within and among plaques of a single individual and the existence of distinct differences in the organization of amyloid in subjects with different clinical presentations. X-ray microdiffraction was demonstrated to be an effective approach to investigating the structure of amyloid *in situ* within sections of human tissue prepared using standard histological methods. These preparative methods have the potential to alter the structure of amyloid and, indeed, destroy much of the other macromolecular structures present in the tissue. Nevertheless, x-ray scattering from these samples exhibited the properties expected for scattering from amyloid: (i) We observed a 4.7 Å peak in a small percentage of the diffraction patterns taken from tissue shown by neuropathology to have a heavy plaque burden. (ii) This 4.7 Å reflection falls at the scattering angle expected for scattering from amyloid. (iii) The diffraction patterns exhibiting this 4.7 Å peak are clustered into regions of size typical of amyloid plaques. (iv) Clusters of diffraction patterns exhibiting this peak are also observed at the margins of vasculature in some samples. (v) The 4.7 Å peak has a doublet character that has been reported extensively in studies of amyloid^33^,^38^. (vi) In experiments involving Congo Red staining, diffraction patterns exhibiting the 4.7 Å peak are limited to the stained regions of tissue. (vii) The orientation of amyloid as indicated by the 4.7 Å peak is not random, but follows patterns consistent with those that have been reported using optical methods^43^. (vii) Diffraction patterns exhibiting a 4.7 Å peak also generally have small-angle and 10 Å scatter consistent with that expected for scattering from amyloid. On these bases, we conclude that our observations have identified and produced information relevant to the characterization of amyloid fibrils embedded in thin sections of human brain tissue.

Orientation of the fibrils appears to be correlated over 10’s of microns, is preferentially tangential to the edge of blood vessels and relatively disorganized in smaller plaques. Congo red staining enhanced the detectability of orientation but did not appear to alter the general patterns observed. In some cases, the orientation at the center of a plaque appeared less well defined than along the periphery. This may be due to intrinsic disorder within the core; to multiple preferred directions occurring through the thickness of the section; or to a preferred orientation perpendicular to the section (and thereby parallel to the incident x-ray beam and unobservable in these experiments). There appears to be longer-range correlation in fibril orientation in larger plaques than in smaller. This might suggest that fibrils progressively pack tighter and with longer-range spatial correlations as plaques mature.

Within a single section of tissue from one AD subject, we observed both perivascular amyloid deposits and plaques not associated with the vasculature. No distinct differences in fibril structure or structural variations were observed between the fibrils in these two environments. It is well known that plaques can exhibit a variety of morphologies that have been classified by light microscopy^45^ - diffuse, fibrillar or dense-cored. We have not, as of yet, collected an adequate number of field of views to confidently assign the plaques observed here to the commonly described morphological classes.

The systematic variation in the shape of the 4.7 Å peak reported here may reflect a systematic variation in fibril structure, perhaps related to packing, assembly or processing of fibrils within plaques, or to distinct differences in quaternary organization of peptides within fibrils. Detailed interpretation will require comprehensive modeling. However, basic principles of helical diffraction theory provide some guidance as to the meaning of the observed shifts in intensity. The doublet form of the peak arises from a combination of the intensity distribution along the 4.7 Å layer line and the significant disorientation common to virtually all diffraction patterns from these tissues. The lower angle peak (~4.75 Å) corresponds to a meridional or near meridional intensity peak on the 4.7 Å layer line. The wider angle peak (4.65 Å) corresponds to an off-meridional peak. Every peak in an x-ray pattern can be thought of as arising from a periodic variation of electron density that acts like a diffraction grating within the sample. A peak on the meridian corresponds to scattering from a diffraction grating with structural elements perpendicular to the fibril axis and periodic along the axis. In a cross-β structure like amyloid, these elements are β-strands and, they pack approximately 4.7 Å apart, constituting the structural elements that give rise to the 4.7 Å layer line. When most of that intensity is meridional, it suggests that the β-strands are perpendicular or nearly perpendicular to the fibril axis. If the β-strands tilt relative to the fibril axis (become less perpendicular), this has the effect of shifting intensity off-meridian. The effect that tiling of β-strands has on scattering from amyloid fibrils has been discussed^36^ (Fig. 4 in Jahn *et al*.^36^). A tilt of somewhat less than 15^o^ would be consistent with off meridional intensity at about the position of the ~4.65 Å peak. When spread around the center of the diffraction pattern by disorientation of fibrils, this off-meridional intensity will appear at wider angles than the meridional intensities, thus leading to the doublet nature of the 4.7 Å reflection. Consequently, the ratio may provide some measure of the tiling of β-strands in these fibrils.

Congo Red dye appears to enhance the meridional intensity at the expense of the off-meridional. This suggests an enhancement of structural elements perpendicular to the fibril axis, perhaps due to the orientation of the bound CR molecules parallel to the β-strands as has been observed in crystals of amyloid protein pig insulin^46^,^47^.

The variability in the intensity ratio of the 4.75 and 4.65 Å peaks *within* individual plaques suggests that fibrils packed in the dense cores have β-strands more nearly perpendicular to the fibril axis than those on the periphery. This variation may reflect an ensemble of structures related to one another by a low energy structural transition such as a variation in fibril twist that might be influenced by side-to-side packing of fibrils. Variation of the intensity ratio *among* plaques follows the same pattern - the denser plaques exhibiting peak shapes consistent with β-strands more nearly perpendicular to the fibril axis. Diffuse plaques exhibit scattering more similar to the margins of denser plaques.

Plaques in the mismatch case appear to fall into two classes - those with amyloid scattering distinctly different from those in typical AD cases and others that appear similar to the plaques we observed in typical AD. Fibrils in regions where the 4.7 Å peaks are observed to take on an atypical shape (4.65 Å sub-peak stronger than 4.75 Å sub-peak) may have an atypical molecular structure. The distribution of amyloid in plaques giving rise to these atypical reflections appears to be relatively diffuse, for the most part not coalescing into tight cores. Although the pattern of structural variation in the mismatch case is similar to that seen in typical AD, the observed ensemble of peak shapes is shifted beyond that observed in even the most diffuse plaques in typical AD (Fig. 5). It seems possible that the Aβ peptides in these fibrils are organized rather differently from typical AD and the β-strands tilted away from perpendicular by an amount greater than that observed in typical AD.

Deeper characterization of these atypical fibrils may provide insight into their role in disease. If the fibrils themselves are the toxic substance in AD, perhaps a different organization of Aβ peptides decreases their toxicity. Alternately, if Aβ oligomers are the actively toxic component, do the atypical fibrils in mismatch cases bind to oligomers, sequester them into large, inactive aggregates, and thereby slow or prevent their associated toxicity? Given the marked difference in neuropathology - which includes the presence of amyloid plaques and tangles but relative preservation of neurons and synaptic markers^13^ - it is as of yet difficult to assess the significance of these observations.

These results document polymorphisms in amyloid fibril structure within individual plaques, among plaques in the same tissue and in tissue derived from subjects with different clinical histories:

The observation of polymorphism in amyloid fibril structure within individual plaques complements extensive *in vitro* observations of fibril polymorphisms^14^,^15^,^16^,^17^,^18^,^19^,^20^,^21^,^22^,^23^,^48^ and makes an initial linkage between polymorphisms in fibril structure with their positions within plaques. The systematic variation between the core and periphery suggests a process in which fibril remodeling occurs during plaque formation and growth. These differences may reflect co-existence of multiple ‘strains’ of amyloid within a single plaque, or they may simply be due to changes in the twist of fibrils driven by progressive packing of fibrils during plaque growth or maturation. It is possible that these differences represent an ensemble of structures within a single ‘strain’ rather than evidence of distinct strains.

We show in two cases that plaques harboring distinct ensembles of amyloid structures coexist within a single tissue section. This could be evidence for multiple strains of amyloid within a single brain or could reflect different stages of plaque maturation. The existence of multiple strains would contradict the suggestion that individual brains may accommodate a single predominant structure formed by self-propagation following a single seeding event^12^,^26^. Such a mechanism has been suggested as a primary driver of disease progression^49^,^50^. The potential role of plaque maturation on disease progression is unknown.

We also demonstrate that tissue derived from subjects with different clinical histories may harbor different ensembles of fibrillar structures. This observation appears to support the conclusion of Lu *et al*.^12^ that distinct amyloid structures may be associated with different disease states.

In summary, these results suggest that each brain accommodates an ensemble of amyloid structures, that the ensemble may exhibit significant variation among subjects and that the nature of the ensemble and its constituent fibrils may determine clinical outcome.

### Experimental Procedures

#### Tissue samples

Human brain tissue was obtained through the Massachusetts Alzheimer’s Disease Research Center that maintains a brain tissue bank at the Massachusetts General Hospital (MGH). The brains collected by MADRC are handled, dissected, and stored in a uniform fashion. Brains are divided in half with one half fixed, processed in paraffin for neuropathological analysis and immunohistochemical studies and the other half generally snap-frozen in coronal slices. Age matched controls were between 85 and 95 years of age and showed no overt signs of dementia at time of death. AD subjects were chosen for high plaque burden as were regions of the brain for analyses. The samples described in detail here were all from the amygdala. Thin 18 μ sections of fixed, paraffin-embedded tissue were cut and captured on 12 μ thick mica sheets. They were then de-paraffinized by heating to 75^o^ C for a minimum of 12 hours and then washed for 5 minutes each with 100% xylene, 100% ethanol, 10% ethanol and then water. Immediately before or after a section was cut for x-ray analysis, a serial section was cut to 5 μ thickness, captured on a glass microscope slide, processed for histological examination and immunostained with 10D5 antibody (1:50, Elan Pharmaceuticals) to locate amyloid plaques. These serial sections made possible location of regions with high probability of containing plaques. Small blood vessels crossing serial sections served as useful fiducial marks for locating these regions in the sections to be studied with x-rays.

#### Microdiffraction data

X-ray diffraction data were collected at beamline 23ID-B at the APS using the scanning microdiffraction capability developed for use in macromolecular crystallography^51^. Samples were aligned using a co-axial optical microscope with a hole down the optical axis to accommodate the x-ray beam thereby allowing precision alignment of the beam to select positions on the sample. A 5 μ beam size was used and samples were stepped along a 5 μ grid with a wide-angle diffraction pattern collected at each grid point. A specimen-to-detector distance of 300 mm was used with an x-ray wavelength 1.033 Å (x-ray energy of 12 keV). The exposure time was 2 seconds. Patterns were recorded with a MAR300 detector with 4096 × 4096 pixels in an area of 30.00 cm × 30.00 cm. Pixel size is 73.24 micron. A typical scan consisted of 2500 diffraction patterns covering a 250 × 250 μ field of view.

Data were analyzed using a suite of programs developed locally^52^ and designed specifically for identifying and quantitating specific features in diffraction patterns (available upon request). These programs included software for subtracting scatter from mica and air; circular averaging of x-ray data; identification of the 4.7 Å peak; integration of the intensity of the 4.7 Å peak; estimation of the intensity of diffuse scattering in the SAXS and 4.7 Å regimes; calculation of the azimuthal distribution of intensity in each diffraction pattern; identification and quantitation of the degree and direction of orientation of the 4.7 Å peak, when present. Ratio of the intensity in the 4.75 and 4.65 Å peaks was estimated by fitting the observed intensity in the 4.7 Å region to a triplet of peaks - a second order polynomial to fit the slowly varying diffuse scattering background and a pair of Gaussians centered at approximately 4.75 and 4.65 Å spacings. The intensity was better accounted for by allowing the positions of these two Gaussians to vary over a small range of 1/d +/− 0.0003 Å^−1^. Regions in which the estimated peak heights were < 3σ were deemed to be devoid of amyloid (where σ is the standard deviation of signal after subtraction of diffuse scatter. Scatter plots of the intensities of the two sub-peaks for 6 scans are included in the Supplementary Materials.

## Additional Information

**How to cite this article**: Liu, J. *et al*. Amyloid structure exhibits polymorphism on multiple length scales in human brain tissue. *Sci. Rep.* **6**, 33079; doi: 10.1038/srep33079 (2016).

## Supplementary Material

Supplementary Information

## Figures and Tables

**Table 1:** Tabulation of 22,800 diffraction patterns collected including number in which a sharp 4.7 Å reflection was observed and the total number of patterns in each scan.

Sample	# diffraction patterns with observable 4.7 Å reflection	average ratio I(4.75)/I(4.65)	average ratio atan(I(4.75)/I(4.65))
AD subject #1 with CR	367/2500	2.38	64.5°
AD subject #1	239/2500	0.95	41.6°
AD subject #2	21/2500	2.21	59.4°
AD subject #3	12/2800	2.18	58.4°
mismatch scan #1	25/2500	0.69	33.6°
mismatch scan #2	102/2500	0.82	37.8°
age-matched control with CR	2/2500	0.47	24.8°
age-matched control	0/2500	—	—
Pick’s subject	4/2500	0.71	30.0°

Also listed are the average ratio of intensity of the 4.75 Å reflection divided by the intensity of the 4.65 Å reflection and atan of that ratio.
