# Supplementary material for: Amyloid structure exhibits polymorphism on multiple length scales in human brain tissue
Source: Sci Rep. 2016 Sep 15;6:33079. doi: 10.1038/srep33079 (PMC5024092; doi:10.1038/srep33079)

Supplementary Material for:

Amyloid structure exhibits polymorphism on multiple length scales in human brain tissue

Jiliang Liu[1], Isabel Costantino[2], Nagarajan Venugopalan[3], Robert F. Fischetti[3], Bradley T. Hyman[2], Matthew P. Frosch[2], Teresa Gomez-Isla[2], and Lee Makowski*[1]

1. Department of Bioengineering, Northeastern University.
2. Massachusetts Alzheimer's Disease Research Center, Massachusetts General Hospital.
3. GM/CA CAT, Advanced Photon Source, Argonne National Laboratory.

Contact Information:

*Corresponding Author:
Lee Makowski l.makowski@neu.edu

1. X-ray microdiffraction from Congo red stained section

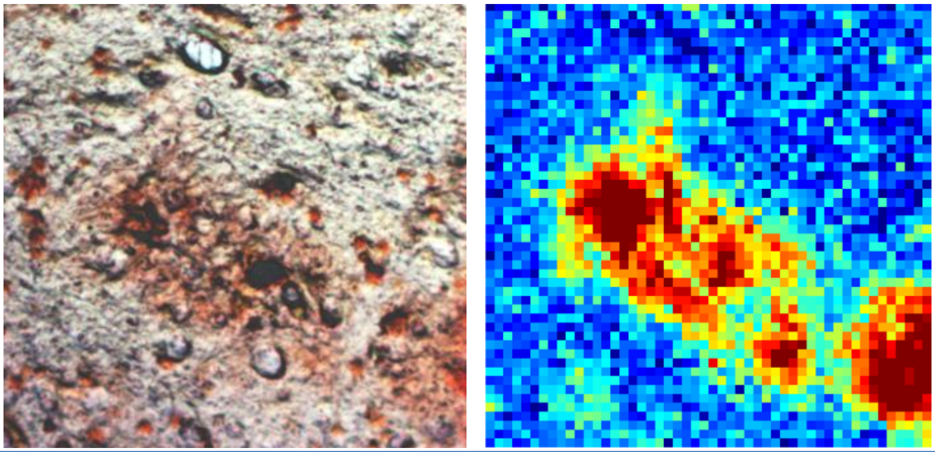

X-ray microdiffraction from Congo Red stained section: left: optical micrograph of stained tissue section. right: mapping of the intensity of the sharp 4.7 Å peak in 2500 diffraction patterns taken over the same 250x250 μ field of view. The regions of strongest 4.7 Å scatter correspond to those regions that contain amyloid as indicated by staining with Congo Red.

2. AD Subject #2 - additional field of view

In this field of view there was a single region that exhibited sharp 4.7 Å scatter indicative of amyloid. A number of places exhibited a sharp 4.15 Å peak diagnostic for the presence of residual paraffin.

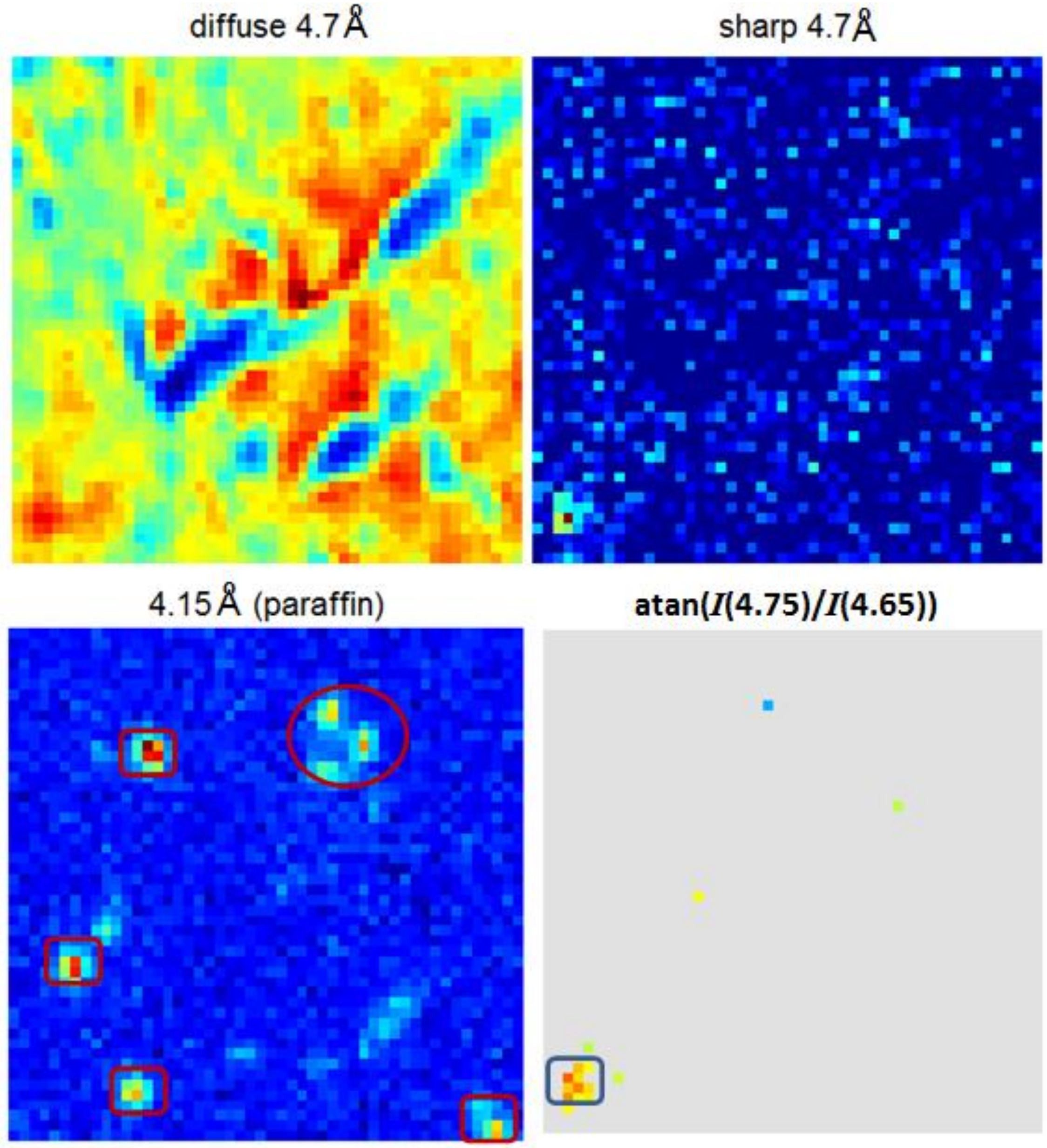

3. AD Subject #3 - additional field of view

This field of view is dominated by a blood vessel travelling through the tissue in the plane of the section. Some diffuse amyloid appears to be present near the bottom center.

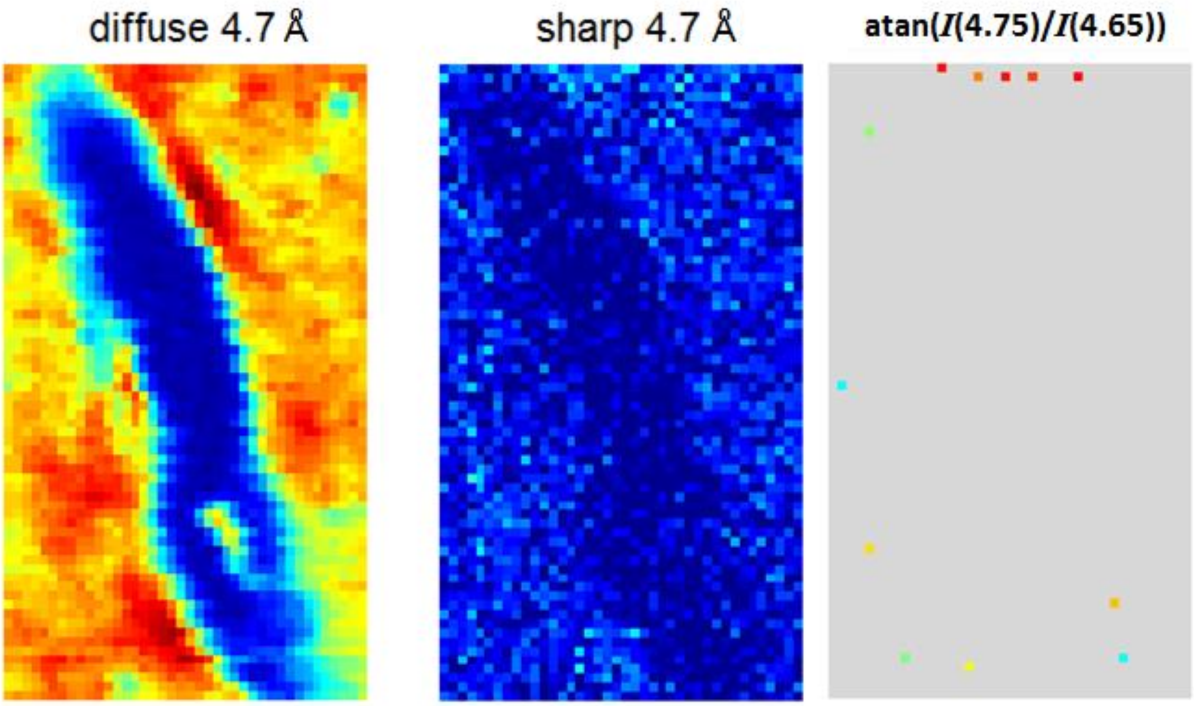

4. Mismatch Subject - additional field of view

Two small amyloid-containing areas are observed near the bottom of this field of view, apparently as a part of a diffuse deposit.

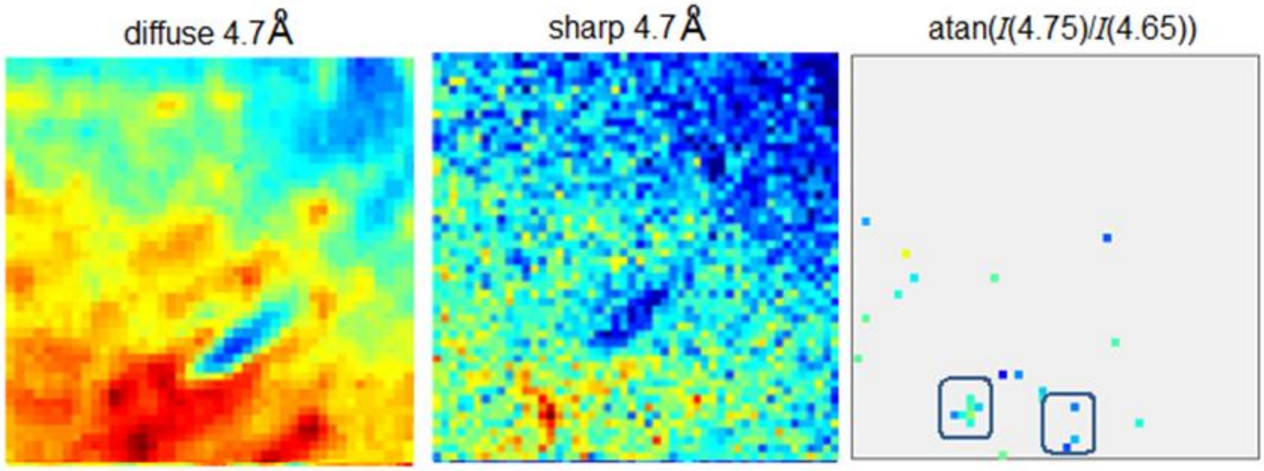

5. Aged-matched control - additional field of view

In this field of view there was no sharp 4.7 Å scatter indicative of amyloid. A number of places exhibited a sharp 4.15 Å peak diagnostic for the presence of residual paraffin.

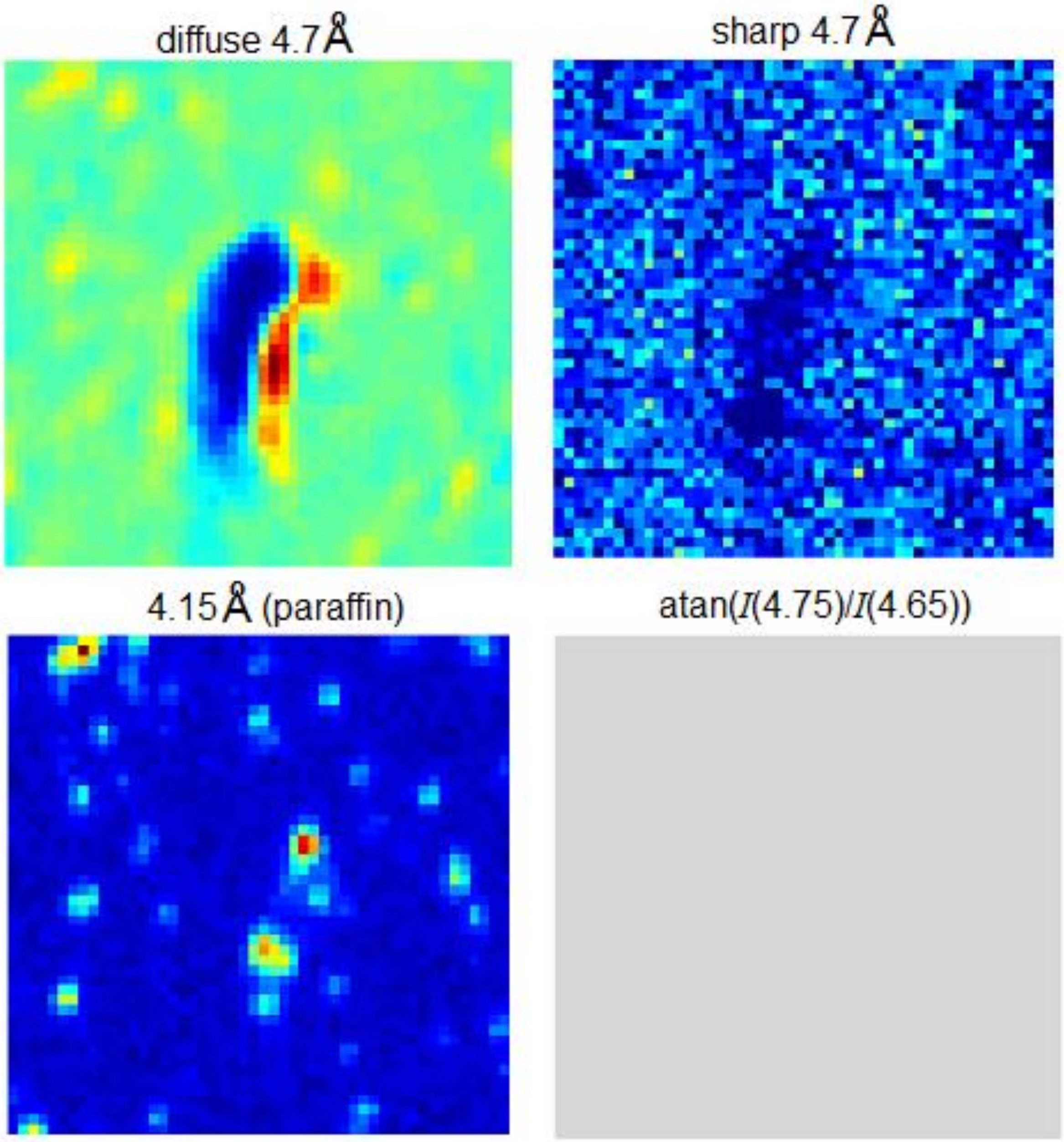

6. Picks Subject - additional field of view

In this field of view there was scant evidence of diffuse amyloid at one or two places. A number of regions exhibited a sharp 4.15 Å peak diagnostic for the presence of significant amounts of residual paraffin.

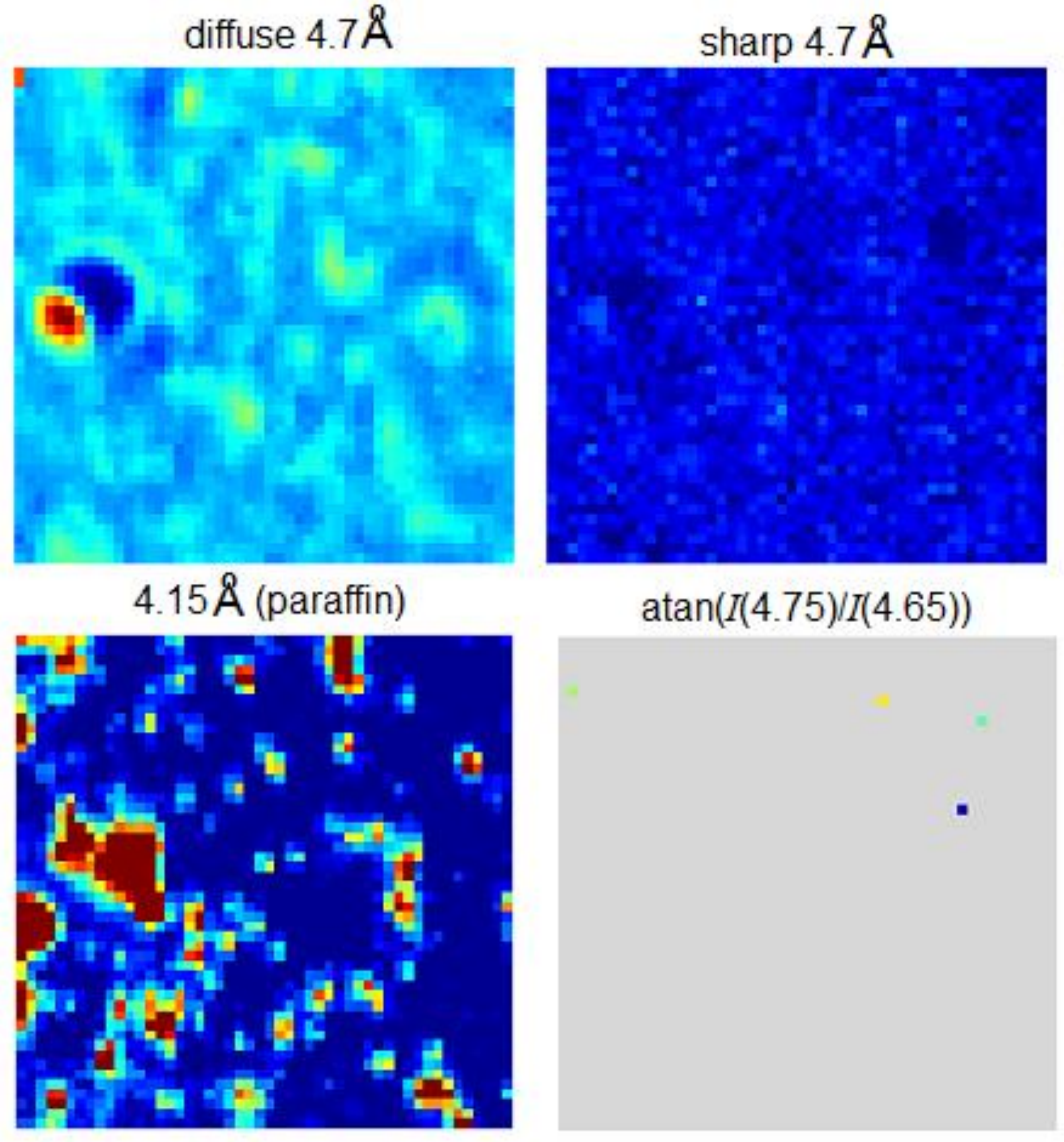

7. Distribution of sub-peak heights

Plotting height of the 4.75 Å peak against height of the 4.65 Å peak, resulted in scatter plots that provided significant distinction among the subjects studied. Automatic fitting of the 4.7 Å peak to two Gaussians was used to estimate the sub-peak heights for all diffraction patterns. Here, the intensity of the 4.75 Å peak (vertical axis) is plotted as a function of the 4.65 Å peak (horizontal axis) for all 2500 diffraction patterns in six scans taken from five subjects. Only those points falling outside the indicated circles correspond to peaks that are considered to be above noise levels (3σ). Most diffraction patterns do not show statistically significant 4.7 Å intensity. Lines indicate average angle (ratio of 4.75 to 4.65) for those patterns exhibiting signal above noise levels.

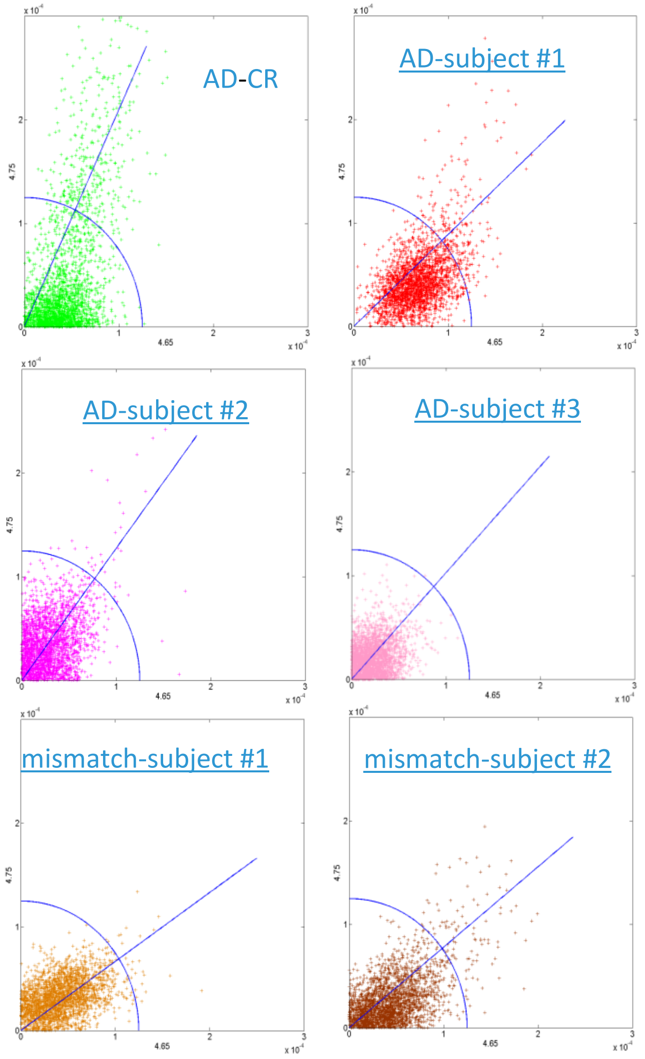

Supplement: Supplementary Information [file srep33079-s1.pdf]
